# Catching viral breast cancer

**DOI:** 10.1186/s13027-021-00366-3

**Published:** 2021-06-10

**Authors:** James S. Lawson, Wendy K. Glenn

**Affiliations:** School of Biotechnology and Biomolecular Sciences, University of New South Wales, Sydney, Australia

**Keywords:** Breast cancer, Multiple virus, Mouse mammary tumour virus, Human papilloma virus, Epstein Barr virus, Bovine leukemia virus, Causation, Evidence

## Abstract

**Abstract:**

We have considered viruses and their contribution to breast cancer.

**Mouse mammary tumour virus:**

The prevalence of mouse mammary tumour virus (MMTV) is 15-fold higher in human breast cancer than in normal and benign human breast tissue controls. Saliva is the most plausible means of transmission. MMTV has been identified in dogs, cats, monkeys, mice and rats. The causal mechanisms include insertional oncogenesis and mutations in the protective enzyme ABOBEC3B.

**Human papilloma virus:**

The prevalence of high risk human papilloma viruses (HPV) is frequently six fold higher in breast cancer than in normal and benign breast tissue controls. Women who develop HPV associated cervical cancer are at higher than normal risk of developing HPV associated breast cancer. Koilocytes have been identified in breast cancers which is an indication of HPV oncogenicity. The causal mechanisms of HPVs in breast cancer appear to differ from cervical cancer. Sexual activity is the most common form of HPV transmission. HPVs are probably transmitted from the cervix to the breast by circulating extra cellular vesicles.

**Epstein Barr virus:**

The prevalence of Epstein Barr virus (EBV) is five fold higher in breast cancer than in normal and benign breast tissue controls. EBV is mostly transmitted from person to person via saliva. EBV infection predisposes breast epithelial cells to malignant transformation through activation of HER2/HER3 signalling cascades. EBV EBNA genes contribute to tumour growth and metastasis and have the ability to affect the mesenchymal transition of cells.

**Bovine leukemia virus:**

Bovine leukemia virus (BLV) infects beef and dairy cattle and leads to various cancers. The prevalence of BLV is double in human breast cancers compared to controls. Breast cancer is more prevalent in red meat eating and cow’s milk consuming populations. BLV may be transmitted to humans from cattle by the consumption of red meat and cow’s milk.

**Conclusion:**

The evidence that MMTV, high risk HPVs and EBVs have causal roles in human breast cancer is compelling. The evidence with respect to BLV is more limited but it is likely to also have a causal role in human breast cancer.

**Supplementary Information:**

The online version contains supplementary material available at 10.1186/s13027-021-00366-3.

## Background

The purpose of this review is to inform the scientific and general community about the causal role of oncogenic viruses in human breast cancer. The authors conducted a review of these viruses and breast cancer in 2017 [1]. New evidence has confirmed the role of viruses as the probable underlying cause of breast cancer. This new evidence includes the identification of mouse mammary tumour virus (MMTV) in sporadic breast cancers but not in BRCA1 breast cancers [2]. This indicates that MMTV is an external not genetic causal factor. MMTV has been identified in the teeth of 4500 year old Mediterranean skeletons [3]. This is an indication that MMTV has been involved with humans for millenia. New case control studies have confirmed that the prevalence of human papilloma viruses (HPVs) in breast cancer is significantly higher than in normal and benign breast tissue controls [4, 5]. HPVs have been identified in circulating vesicles [6]. This suggests a means of transmission of HPVs from genital areas to the breasts. There is new evidence that Epstein Barr viruses enhance the role of HPVs in breast cancer including the invasive capacity of breast cancer cells [7–9]. New case control studies have confirmed the probable role of bovine leukemia virus (BLV) in human breast cancer [10–12]. BLV has been identified in human blood which suggests a means of internal transmission of this virus to breasts [13].

This new evidence confirms the probable causal roles of mouse mammary tumour virus (MMTV), high risk human papilloma virus (HPVs), Epstein Barr virus (EBV) and bovine leukemia virus (BLV) in human breast cancer.

## Methods

Reviews of case control studies of viruses and breast cancer have been previously conducted. These include (i) MMTV by Lawson & Glenn 2019 [14], (ii) HPVs by Ren et al. (2019) [15], (iii) EBV Lawson et al. 2018 [1], (iv) BLV by Buehring & Sans 2019 [16]. The details in these case control studies have been included as Supplementary Tables. Reviews of case control studies do not consider the range of additional evidence required to assess causality. This additional evidence is considered in this review.

We have used an extended version of the classic A. Bradford Hill causal criteria to assess the evidence concerning the role of these oncogenic viruses [17]. The Hill causal criteria include – identification of the causal pathogen, strength of association between the pathogen and the cancer, consistency, temporality, specificity, biological gradient, plausibility, coherence, experiment, and analogy [17]. We have included additional criteria to consider means of transmission and oncogenic mechanisms.

Statistics: The Chi-square non-parametric test was applied using the SPSS system.

### Outcomes

#### Multiple oncogenic viruses in breast cancer

Multiple oncogenic viruses have been identified in breast cancers in the same patients. Mouse mammary tumour -like viruses, high risk human papilloma viruses, Epstein Barr virus and bovine leukemia virus have each been identified in the same breast cancers in the same patient [18]. These same viruses have been identified in benign breast tissues 1 to 11 years before the development of same virus positive breast cancers in the same patients [18, 19]. Cytomegaloviruses and other viruses have also been identified in breast cancers but the evidence indicating they may have a causal role is limited [20].

It is possible that there may be interaction between HPVs and Epstein Barr viruses leading to breast cancer [7]. There is limited experimental evidence that HPVs can influence the oncogenicity of EBVs [21].

The complexity of the interaction between oncogenic viruses in breast and other cancers is illustrated by the experimental evidence that HPVs can adversely influence the anti-viral enzyme Apolipoprotein B Editing Enzyme (APOBEC3B) which in turn can increase the risk of MMTV associated breast cancer [22–24]. EBV has also been shown to adversely affect APOBEC3B [25].

Both HPVs and EBVs have been found to co- present in 40% of blood donors from the Middle East suggesting a possible route to the breast for both viruses [9].

## Mouse mammary tumour virus

### History

The development of the evidence that MMTV has a likely role in human breast cancer has been extraordinarily difficult and has taken over 80 years of intense work by hundreds of scientists.

The story began when John Bittner, a scientist working at the Jackson laboratories in the United States during the 1930 economic depression years, identified what he called a “milk factor” in mouse mothers with breast cancer which was transmitted via mouse milk to mouse pups. When these mouse pups grew to adulthood they developed breast cancer. It was not until after the second world war years that this “milk factor” was identified as a cancer causing virus which became known as mouse mammary tumour virus. In 1972 Axel et al. demonstrated for the first time that MMTV was present in human breast cancers but not in normal and benign breast tissues. It was obvious this virus may also cause breast cancer in humans but it was impossible to develop scientific proof despite enormous research efforts during the President Richard Nixon 1970’s “War on cancer”.

A breakthrough in technology was required and in 1983 Kary Mullis of California developed polymerase chain reaction technology (PCR). In 1995 Beatriz Pogo and her colleagues, working in New York, were able to use PCR technology to demonstrate that MMTV or a virus almost identical to MMTV, was present in many human breast cancers but rarely in normal human breast tissues. Similar observations were soon made in other countries including Australia. This was strong evidence that MMTV may have a role in human breast cancer.

Further advances were made. Working in Vienna, Stanislav Indik and colleagues showed that MMTV could infect human breast cells [26]. MMTV was identified in human milk in normal lactating Australian women [27]. Nartey et al. demonstrated that MMTV was more common in human milk from US women with breast cancer [28]. Mazzanti et al. of Pisa, Italy, showed that MMTV was frequently present in the saliva of women with breast cancer [29]. Lawson and Glenn demonstrated that MMTV, HPV, EBV and BLV were present in normal and benign breast tissues before the development 1 to 11 years later of same virus positive breast cancer in the same women [18]. This is an important causal criteria. Finally the Bevilacqua group in Pisa demonstrated that MMTV was present in 30% of sporadic breast cancers as compared to 4% of hereditary breast cancers (thereby creating a natural control series of breast cancers) which is an indication that MMTV has a probable causal role [2]. MMTV gene sequences in dental material from citizens of the copper age some 4500 years ago, have been identified by the Bevilacqua group in Pisa [3].

### Identification and strength of association between MMTV and human breast cancer

#### MMTV structure

MMTV is a retrovirus with a 8 kbase genome core surrounded by a capsid which is bound to the outside envelope by a matrix protein. The genome encodes 7 genes, one of which – Sag, is involved in mammary infection and subsequent tumorigenesis.

#### Identification

MMTV-like gene sequences have been repeatedly identified in breast cancers by hybridisation techniques, standard liquid PCR, in situ PCR, microdissected PCR and whole genome sequencing. The use of different techniques for the identification of MMTV in human breast cancer is important as this overcomes concern about contamination and false positive outcomes. Concern about contamination by rodent sourced MMTV has been overcome by the absence of mouse mitochondrial DNA sequences in breast cancer specimens. The MMTV gene sequence identified in human breast cancers is 95 to 99% homologous with MMTV identified in mouse mammary tumours [30].

The identification of MMTV-like gene sequences in human breast cancer varies between 6 to 78% with the most common prevalence approximately 40% compared to negligible identification of MMTV in normal and benign human breast tissues.

The first case control study of MMTV and human breast cancer was by Axel et al. in 1972.

[31]. Using molecular hybridisation techniques they identified MMTV in 18 (62%) of 29 breast cancers but could not identify MMTV in 13 normal and benign breast tissues.

Based on 22 case control studies as listed in Supplementary Table 1, the prevalence of MMTV was 750 (27.6%) of 2715 in human breast cancers as compared to 131 (7.8%) of 1671 in normal and benign breast tissue controls (*p* = 0.001).

#### Consistency

MMTV-like gene sequences have been identified in human breast cancer in a wide range of Western and Asian countries [1]. The positive identification of MMTV in breast cancer is consistently higher in Western than Asian countries – 40% compared to 8.5%.

MMTV antibodies in the serum of women with breast cancer is 5 fold higher than normal controls [1]. Women exposed to MMTV in laboratories have been shown to develop a serological response [32].

#### Specificity

MMTV is not specific for human breast cancer. MMTV has been identified in many human cancers [33].

#### Temporality (does the causal agent precede a specific disease)

MMTV has been identified in normal and benign human breast tissues 1 to 11 years before the development of MMTV positive breast cancer in the same women [18, 34].

#### Biological gradient

The MMTV viral load increases as breast cancer progresses but falls in late stage cancer. The fall in viral load appears to be due to the breakdown of cell physiology [35, 36].

#### Plausibility (oncogenic capacity of the infectious agent)

MMTV is the proven cause of mammary cancers in feral mice. MMTV has also been identified in mammary tumours of dogs and cats [37].

#### Experimental evidence

MMTV can infect human breast cells and randomly integrates into the human genome [38]. This indicates the possibility of an infectious transmission of MMTV in humans.

#### Transformation

In 2005 Katz et al. found proteins expressed by the MMTV envelope gene were capable of malignantly transforming normal human breast epithelial cells and in 2012, Feldman et al. found the MMTV envelope protein p14 may play a role in altering the oncogenic potential of MMTV-infected cells [39, 40].

#### Transmission

Transmission of MMTV in humans may be by saliva, human milk, dogs and contaminated food cereals.

MMTV gene sequences have been identified in human saliva [29]. MMTV sequences are present in 27% of normal children, 11% of normal adults and 57% of women with breast cancer [29]. A mother, father and daughter living together all developed MMTV positive breast cancer [41]. These observations suggest that saliva is probably the main means of transmission of MMTV.

MMTV has been identified in human milk [27, 28]. The presence of MMTV in human milk is associated with a higher than normal risk of breast cancer.

As MMTV has been identified in dogs it is possible that MMTV is transmitted via dog saliva [42].

Transmission is also possible via mouse and rat faecal material. In many countries up to 1% in weight of cereals may legally consist of rodent faecal material [43].

#### Oncogenic mechanisms

Insertional oncogenesis. In experimental mice infected by MMTV the virus is inserted into the mouse genome. In response proto-oncogenes such as Wnt are activated and later lead to mammary cancers [44]. In MMTV positive human breast cancers Wnt-1 expression is high [45]. MMTV insertion sites and associated gene mutations have been identified in many human breast cancers [46]. These observations suggest that the causal role of MMTV in human breast cancer may be the same as its role in mice.

Viruses and APOBEC enzymes. Recent studies have demonstrated that MMTV is more complicated than insertional oncogenesis (as in mice) than previously thought. Of particular interest is the battle between retroviruses such as MMTV and APOBEC3 genes [47]. The APOBEC3 family of genes and proteins in mammals are protective against retroviruses. Other viruses including human immunodeficiency virus (HIV the cause of AIDS), encode viral proteins to overcome the antiviral effects of the APOBEC3 proteins of their hosts. Recent studies have revealed that the acquisition of an anti-APOBEC3 ability by lentiviruses is a key step in achieving successful cross-species transmission. This is relevant to the possible mouse to human transmission of MMTV. Such transmission may have originally happened many millenia ago [3]. While human papilloma viruses are not lentiviruses, there is evidence that they can damage APOBEC3B and thereby reduce its antiviral protective effects [22, 23]. Abnormal expression of APOBEC3 has been shown to be induced by DNA viruses such as human papilloma virus, specifically in breast cancer [22, 24].

MMTV oncogenic proteins. An additional mechanism is the influence of MMTV envelope proteins which can transform normal human breast cells [39]. The precise mechanism is not known.

#### Conclusions

The evidence meets the extended Hill causal criteria. A causal role for MMTV-like viruses in human breast cancer is probable.

## Human papilloma viruses (HPVs)

### History

Infectious genital warts have been associated with sexual activity since the ancient Greek and Roman eras. In 1842 surgeon Domenico Rigoni-Stern of Padua, suggested that cervical cancer was a sexually transmitted disease because it was more common in married women and prostitutes than virginal women. It was more than one hundred years later that human papilloma viruses emerged as a chief suspect. The key scientific detective was Harald zur Hausen of Germany who in 1976 hypothesised that HPVs may have a role in cervical cancer.

Confirmation of the oncogenic capacity of HPVs in cervical cancer stimulated interest in the possible role of HPVs in other cancers. In 1990 the first tentative steps were by Vilma Band and colleagues who found that HPVs could immortalize normal human breast cells. In the same year Lutz Kowalzick and colleagues identified HPVs in human nipple papillomas Working in Rome, Anna Di Lonardo and colleagues made the first identification of high risk for cancer HPV in breast cancer tumours. They identified HPV type 16 in 29% of a series of breast cancers and metastatic lymph nodes. Their findings were later confirmed in 25 case control studies all of which demonstrated that the prevalence of high risk HPVs was consistently higher in breast cancer than in normal and benign breast controls. The prevalence of high risk HPVs in breast cancer has varied from zero to 74%. The HPV viral load in breast cancer tissues is extremely low as compared to cervical cancer and is therefore difficult to detect. This is the probable reason for the negative and variable outcomes of some studies. This was an indication that the role of HPVs in breast cancer is probably different.

### Identification of human papilloma viruses in breast cancer

#### HPV structure

HPV DNA (about 8 kilo base pairs) is circular, double stranded and is surrounded by a protein capsid coat. A persistent infection, (one that is not cleared by the immune system) of high risk HPVs, can increase the risk of cancer by using oncogenes E6 and E7 which inactivate p53 and pRB.

Since the first identification of HPVs in breast cancers, high risk for cancer HPVs have been identified in breast cancer in many countries [15]. HPV identification in breast cancer tissues has been found in the following countries: Australia, Brazil, China, Egypt, Greece, India, Italy, Iran, Iraq, Japan, Korea, Morocco, Mexico, Spain, Turkey, United Kingdom, United States of America, and Venezuela.

This identification has been by several different methods including standard polymerase chain reaction technology (PCR), in situ PCR, in situ hybridisation, immunohistochemistry and massive parallel sequencing [48]. The prevalence of high risk HPVs in breast cancers substantially varies between studies [15]. High risk for cancer HPV type 16 and 18 dominate in Western women [15]. In addition to HPV types 16 and 18, HPV types 33, 52 and 58 are common in Chinese, Korean, Japanese and Qatari women [15]. These HPV types need qualification. This is because in some studies the PCR primers were specific for particular HPV types and not a review of all HPV types that may have been present in cancer samples. In the studies in which high risk HPVs were identified no correlations have been demonstrated between breast cancer grade, survival, or steroid receptor expression.

The reason for the variations in outcomes is probably due to the technical difficulty of identifying the extremely low HPV viral loads in breast cancer as compared to cervical cancer. In 2008, Khan et al. demonstrated by quantitative PCR that the average viral load of HPV 16 in breast cancers was 5.4 HPV copies per 10^4^ cancer cells as compared to 130,480 in cervical cancer [49].

##### Strength and consistency of association between HPVs and breast cancer

Ren et al. have conducted a recent meta-analysis of these same studies which included 3607 breast cancer cases and 1728 normal and benign breast controls [15]. The odds ratio was 6.2 (*p* = 0.002) for HPV positive breast cancer cases compared to HPV positive controls.

Based on 40 case control studies as listed in Supplementary Table 2, the prevalence of high risk HPVs was 1194 (30.1%) of 3970 breast cancers as compared to 150 (8.4%) of 1796 normal and benign breast tissue controls (*p* = 0.001).

The finding that different methods of identification of HPVs give similar outcomes – namely that the prevalence of HPVs is significantly higher in breast cancer compared to controls, adds to the strength of the evidence that HPVs have a role in breast cancer.

#### Epidemiology

Women who develop HPV associated cervical cancer are at higher than normal risk of developing HPV associated breast cancer [50]. These women are on average 10 years younger at the age of developing breast cancer than other women who develop breast cancer. As younger women are usually more sexually active than older women and are therefore at great risk of sexually transmitted HPV infections, this observation is compatible with a role for HPVs in breast cancer.

#### HPV transformation and oncogenic mechanisms

High-risk HPVs encode proteins, several of which have an oncogenic capacity. HPV proteins are classified as early (E1–E7) or late (L1 and L2). The expression of HPV E6 and E7 proteins lead to malignant changes in normal epithelial cells. HPV E6 proteins degrade p53 (a cancer suppressing gene). HPV E7 enhances viral replication and malignant transformation from normal cells to cancer cells by upregulating Cox-2 which increases inflammation [51]. HPV E5 and E6 act early in transformation and lead to the appearance of koilocytes [52]. Koilocytes are large squamous cells with acentric nuclei surrounded by a halo. The appearance of koilocytes is used by pathologists to indicate early signs of adverse HPV infections of the cervix. Koilocytes have been identified in breast cancers [53].

While several of these HPV oncogenic mechanisms in cervical cancer appear to be involved in breast cancer, additional mechanisms are involved. The details of these additional mechanisms are far from clear and probably involve the antiviral enzyme Apolipoprotein B Enzyme Catalytic 3B (APOBEC3B). HPV infections upregulate and lead to mutations in APOBEC3B [23]. APOBEC3B mutations increase the risk of breast cancer [24].

There is experimental evidence that exposure to HPV E6 and E7 proteins can transform and immortalise normal human breast epithelial cells [54–58]. However as discussed below, it is unlikely that this is the major oncogenic mechanism with respect to HPVs and breast cancer. The main mechanisms are probably both complex and indirect.

HPVs interact with BRCA (BReast CAncer) genes and can lead to mutations which may cause breast cancer [59].

APOBEC3B helps to protect against the harmful effect of viruses [22]. After viral DNA integration, mutations in APOBEC3B can lead to host genome instability and then to breast cancer progression [24]. HPV infections can adversely influence additional members of the APOBEC family (APOBEC3 – A, C, D, E, F, G and H) [25, 60].

HPV type 16 E6 degradation of p53 increases TEAD4 (Transcriptional enhancer factor) levels which can lead to an upregulation of APOBEC3B [61].

HPV16 E7 increases COX-2 expression and promotes the proliferation of breast cancer (COX-2 is an enzyme that increases inflammation) [51].

There are interactions between HPV type 16 oncoproteins and HER 2 (human epidermal growth factor receptor 2 –which promotes the growth of cancer cells) [62].

HPVs and HER2 interact with the E-(epithelial) cadherin complex which is down regulated in cancers [63]. E- cadherins keep cells together.

HPV oncoproteins interact with Epstein Barr virus enhance invasion by breast cancer cells [9].

#### Specificity

HPVs are not specific. Human papilloma viruses are ubiquitous and can infect epithelial and other cell types and lead to many different types of cancer. It is well documented that high risk HPVs are causal in cervical cancer, other ano-genital cancers and head and neck cancers.

#### Temporality HPV infection and subsequent breast cancer time sequence

High risk HPVs of the same type have been identified in benign breast tissues 1 to 11 years before the development of HPV positive breast cancers in the same women [48]. This is an important causal criteria.

#### Transmission

Sexual intercourse is regarded as the primary route of human papillomavirus (HPV) transmission. However, HPVs are stable viruses which can stay on tissue and other surfaces for many days and can probably be transmitted in several different ways. There is evidence which indicates HPVs can be transmitted by non- sexual means from mother to child, fomites (an inanimate object such as a spatula or spoon, that can transfer pathogens to a new host), from health care workers to patients and in an unknown manner to adolescent girls with no sexual experience [64]. There is also evidence that HPVs may be transmitted via saliva and blood [65]. High risk HPVs has been found in (51%) of blood samples from healthy donors [8].

Of much interest is the transmission of HPVs from the place of entry into the body to breast tissues. HPVs are probably transmitted from the cervix to the breast by circulating extra cellular vesicles – also known as exosomes [6, 66]. Extracellular vesicles are extremely small particles (30–100 nm in diameter) which can be expressed or released from cells and biological fluids and can contain proteins and viral nucleic acids. Importantly they can transmit HPV viral nucleic acids from cell to cell [65]. Serum derived extra-cellular vesicles contain HPV DNA which can be transported to breast cells [6].

### Conclusion

Although the underlying mechanisms for a role of HPVs in breast cancer are not clear, the evidence meets all the extended Hill causal criteria.

## Epstein Barr virus (EBV)

### History

In 1957 surgeon Dennis Burkitt, was working in Kampala, the central African capital of Uganda. His attention was drawn to children suffering from horrendous swelling of the face and neck. These children soon died. Following extensive investigations Burkitt and his colleagues identified the disease as acute malignant lymphomas. At a London lecture by Burkitt, Anthony Epstein learned of these experiences and arranged for Burkitt to send him lymphoma specimens. In collaboration with Bert Achong and Yvonne Barr, Epstein identified viral particles in these specimens. These virus particles were later identified as human herpes virus 4 now well known as the cause of infectious mononucleosis and malignant lymphomas. Burkitt’s lymphoma – as the cancer became known, was also linked to malaria. Chronic malaria leads to depression of the immune system. This was an explanation for the geographic location of Burkitt’s lymphoma in Uganda which was confined to the hot, humid, malarial, mosquito ridden areas.

This pioneering work was of universal value because it was the first demonstration that viruses could cause cancer in humans.

### Identification of EBV in human breast tissues

#### EBV structure

Epstein-Barr virus has linear double stranded DNA (approximately 172 kilobase pairs) inside a capsid which is surrounded by a protein rich tegument, which in turn is enveloped by a protein coat. An outer membrane envelope contains glycoproteins which induce viral entry into B-lymphocytes as well as epithelial cells where the virus can remain dormant as a latent infection.

EBV Type 1 is found worldwide. EBV type 2 is also found in Africa and China. (EBV Type 1 differs from Type 2 in the sequence of EBNA genes). EBV type 1 is more dominant in some cancers including leukemia) [67].

Epstein–Barr viruses are the cause of lymphomas and are associated with additional cancers including nasopharyngeal and gastric cancer. Working in London, in 1995, Louise Labreque and colleagues made the first identification of EBV in breast cancers. Various EBV genes have since been repeatedly identified in breast cancer in a wide range of countries. The methods used for the identification of EBVs include standard PCR, in situ PCR with microdissection of the cancer cells, in situ hybridisation and immunohistochemistry (Supplementary Table 3). The use of these various methods offers conclusive evidence of the identification of EBV in breast cancer.

##### Strength and consistency of association between EBVs and breast cancer

EBV positive lymphocytes commonly infiltrate breast cancers. Studies which do not identify breast cancer cells separately from infiltrating lymphocytes are not valid. Therefore a careful assessment of each study is required before inclusion in meta-analyses. In a recent meta-analysis of 16 case control studies by Jin et al. 2020 the odds ratio of EBV positive breast cancer compared to normal and benign breast controls was 4.75 (*p* = 0.01) [68] [Jin 2020 70].

*Epstein Barr virus.* Based on 24 case control studies as listed in Supplementary Table 3, the prevalence of Epstein Barr virus was 731 (30.4%) of 2402 breast cancers as compared to 52 (7.5%) of 1044 normal and benign breast tissue controls (*p* = 0.001).

The evidence is consistent that EBVs are significantly more prevalent in breast cancers than controls.

*Temporality:* EBV infection and subsequent breast cancer time sequence.

Epstein–Barr virus gene sequences have been identified in benign breast tissues 1 to 11 years prior to the development of EBV-positive breast cancer [18]. The prior benign and later breast cancer specimens were from the same individual patients.

#### Epidemiology

In economically developed countries EBV associated infectious mononucleosis occurs most commonly among teenagers. This is in contrast to developing countries where EBV infections mainly occur in early childhood. EBV infections in teenagers and young adults is associated with Hodgkins lymphomas. There is a strong correlation between EBV associated Hodgkins lymphoma and breast cancer [69].

#### Transmission

Epstein–Barr virus is mostly transmitted from person to person via saliva [70]. Infection is almost universal in all populations but occurs at a later age among Western people when compared with economically developing communities.

EBV has been found in (61%) blood samples from healthy donors, which might explain transmission through the body [8].

#### Oncogenic mechanisms

Epstein–Barr virus infection predisposes breast epithelial cells to malignant transformation through activation of HER2/HER3 signalling cascades [70]. HER2 and HER3 are two of the cellular oncogenes known to be involved in human breast cancer development and are associated with a relatively poor prognosis.

Several mechanisms have been brought forward for oncogenicity by EBV genes. It is not known if they act separately or in co-operation, but it has been proposed that after infection and malignant transformation EBV presence is not required [71].

1. HER2/HER3, BARF0

Epstein–Barr virus encoded BARF0 infection predisposes breast epithelial cells to malignant transformation through activation of HER2/HER3 signaling cascades [71]. These HER genes are activated by BARF0 [72].

2. APOBEC 3B

EBV infections can lead to APOBEC3 genome instability which inhibits its protective effects against other viruses [25].

3. 2.EBV EBNA GENES

EBV EBNA genes contribute to tumour growth and metastasis and have the ability to affect the mesenchymal transition of cells. Epithelial mesenchymal transition is associated with malignant progression of cells and is associated with cancer metastasis [73–75].

4. EBV EBNA-1 has also been associated with BRCA-1 gene defects which in turn is associated with breast cancer [76].
5. EBV latent membrane proteins (LMP1, LMP2) contribute co-operatively to cancer progression [77, 78].

#### Conclusion

The evidence for a role of EBV in breast cancer meets the extended Hill causal criteria.

## Bovine leukemia virus (BLV)

### History

A lymphoma type disease was first recognised in cattle as early as 1871. However it was not until 1969, when Janice Miller and Kenneth Gillette of the US identified virus like particles in cattle lymphosarcoma, which is the underlying cause of what became known as bovine leukemia virus. There was obvious concern that this virus could also cause cancer in humans. Unfortunately, in the same year, 1969, the US National Animal Monitoring System declared “BLV is not transmissible to humans and no human disease has ever been attributed to BLV” [79]. Fortunately due to the development of sensitive western blotting techniques Gertrude Buehring and her colleagues at the University of California at Berkeley were able to identify BLV antibodies in human blood serum [80].

## Identification of BLVs in human breast cancer

### BLV structure

Bovine leukemia virus is an oncogenic retrovirus capable of integrating into a host’s DNA causing a lifetime infection. The RNA is enclosed within a capsid, which in turn is surrounded with a transmembrane with glycoproteins attached. The genome (8.7 kilobases) contains normal retroviral genes as well as a regulatory region encoding Tax and Rex which are capable of affecting host DNA repair and growth control.

In 2014 Buehring and her colleagues, using PCR and immunohistochemistry identified BLV in human breast cancers in US women [81]. They identified BLV in 44% of breast cancers. BLV has since been identified in breast cancers in women from Argentina, Columbia and Brazil. BLV has been detected in 38% of leukocytes in blood from women without breast cancer [13].

BLV was not identified in breast cancers in European and Chinese women [82, 83]. However Gillet et al. used massive parallel sequencing to seek to identify BLV. This technology has been shown to not necessarily identify low viral loads or viruses that have not integrated into the cancer genome. Zhang et al. used techniques designed for use in cattle not humans and there is doubt about the validity of the outcomes [83].

#### Strength and consistency of association between BLVs and human breast cancer

The identification of BLV virus in breast cancer has been confirmed. Based on 8 case control studies the prevalence of BLV was 287 (43.4%) of 662 breast cancers compared to 131 (24.0%) of 545 normal and benign breast controls (*p* = 0.001). With two exceptions the prevalence of BLV is significantly higher in breast cancers as compared to normal controls (Supplementary Table 4).

Of special interest is a recent study of BLV in human breast cancers in south eastern Brazil, where the people traditionally ingest raw (unpasteurized) milk and cheese. The prevalence of BLV in the dairy cattle present in more than 90% of the animals. BLV was identified in 96% of human breast cancers as compared to 59% of normal breast controls [12].

#### Epidemiology, transmission

BLV infects cattle in the Americas, some parts of Europe and Asia plus the Middle East. In cattle BLV is mainly located in lymphocytes and mammary epithelial cells which can exfoliate into milk. Women with lactose intolerance are at decreased risk of breast cancer [84]. Breast cancer is more prevalent in red meat eating and cow’s milk consuming populations [85]. Accordingly it can be postulated that BLV can be transmitted to humans from cattle by the consumption of red meat and cow’s milk.

BLV has also been found in human female blood samples. The implication is not known but could indicate a means of internal BLV transmission [10, 13].

*Temporality:* BLV infection and subsequent breast cancer time sequence.

In a study of Australian women with breast cancer BLV was already present in benign breast tissue years 3–10 years before BLV positive malignancy was diagnosed [19]. This observation is consistent with the causal criteria of a prior infection before the development of same pathogen related cancer.

#### Conclusion

It is likely that BLV has a causal role in some human breast cancers but additional evidence is required before any conclusions can be made.

## Discussion

The evidence that mouse mammary tumour virus, human papilloma viruses and Epstein Bar virus may be singly or jointly involved in breast oncogenesis is compelling. The prevalence of each of these oncoviruses is significantly and consistently higher in breast cancer than in normal and benign breast controls. In addition the evidence is sound that each of these viruses meets the Hill evidentiary causal criteria which includes – identification of the causal pathogen, strength of association between the pathogen and the cancer, consistency, temporality, specificity, biological gradient, plausibility, coherence, and analogy. The underlying causal mechanisms are not clear.

There is also sound evidence that multiple oncogenic viruses can be present in breast cancers in the same patients [1]. While each of these viruses are oncogenic and can cause cancer when acting alone, there is evidence that there is probable interaction between several of these viruses, in particular human papilloma viruses and Epstein Barr virus [7–9]. There is also evidence that human papilloma viruses can adversely influence the viral protective enzymes of the ABOBEC family which can increase the risk of viral associated breast cancers such as mouse mammary tumour virus [22–24].

The evidence for a role of bovine leukemia virus in breast cancer is more limited but probable.

## Prevention

Mouse mammary tumour virus: studies conducted several decades ago on experimental mice indicate the possibility of developing preventive vaccines [86]. More recently Braitbard et al. working in Israel have shown the possibility of MMTV derived proteins used to prevent and treat MMTV breast infections [87].

There are safe and effective vaccines available for the control of HPV infections [88].

It has not been possible to develop vaccines for the control of Epstein-Barr infections.

Although the evidence for a causal role for bovine leukemia virus is not conclusive, it is prudent to encourage preventive measures. These include the avoidance of uncooked meat and unpasteurised milk. A much more difficult measure is the culling of infected cattle. This has been achieved for commercial motives in Australia and parts of Europe but not in the Americas or Asia.

## Conclusion

Each of these four viruses has established oncogenic potential. The evidence that MMTV, high risk HPVs and EBVs have causal roles in human breast cancer is compelling. The evidence with respect to BLV is more limited but it is likely to also have a causal role in human breast cancer.

## Supplementary Information

**Additional file 1: Table S1.** Identification of MMTV sequences in breast cancer (Case control studies). DCIS – ductal carcinoma in situ; IDC – invasive ductal carcinoma; ns = not significant at 0.05 level. **Table S2.** identification of high risk for cancer human papilloma virus in breast cancers and controls (case control studies). The prevalence of high risk HPV is consistently higher in all studies of breast cancers as compared to controls. The difference is statistically significant for 22 of 25 studies. ns = not significant at 0.05 level. **Table S3.** Case control studies Epstein Barr virus and breast cancer. PCR = polymerase chain reaction, IHC = immunohistochemistry, ISH = in situ hybridisation, ns = not significant at 0.05 level. **Table S4.** Identification of Bovine leukemia virus in human breast cancer. ns = not significant at 0.05 level.

## Data Availability

All data is available from the authors.

## Abbreviations

MMTV: Mouse mammary tumour virus
HPV: Human papilloma virus
EBV: Epstein Barr virus
BL: Bovine leukemia virus
PCR: Polymerase chain reaction
APOBEC: Apolipoprotein B deaminase enzymes
HER: Human epidermal growth factor receptor proteins
SPSS: Statistical package for social sciences
HPV E6/E7: Human papilloma virus early and late oncoproteins
pRB: Retinoblastoma protein
