# Supplementary material for: Catching viral breast cancer
Source: Infect Agent Cancer. 2021 Jun 10;16:37. doi: 10.1186/s13027-021-00366-3 (PMC8191131; doi:10.1186/s13027-021-00366-3)
Supplement: Supplementary file 1 — Additional file 1: Table S1. Identification of MMTV sequences in breast cancer (Case control studies). DCIS – ductal carcinoma in situ; IDC – invasive ductal carcinoma; ns = not significant at 0.05 level. Table S2. identification of high risk for cancer human papilloma virus in breast cancers and controls (case control studies). The prevalence of high risk HPV is consistently higher in all studies of breast cancers as compared to controls. The difference is statistically significant for 22 of 25 studies. ns = not significant at 0.05 level. Table S3. Case control studies Epstein Barr virus and breast cancer. PCR = polymerase chain reaction, IHC = immunohistochemistry, ISH = in situ hybridisation, ns = not significant at 0.05 level. Table S4. Identification of Bovine leukemia virus in human breast cancer. ns = not significant at 0.05 level. [file 13027_2021_366_MOESM1_ESM.docx]

**Supplementary Tables**

| Study author | Location | Breast cancer pathology | Breast non- cancer pathology  controls | MMTV positive  /total cancer | MMTV positive  /total non-cancer | Statistical significance  P value |
| --- | --- | --- | --- | --- | --- | --- |
| Axel 1972 [1] | US | Invasive | Normal/ benign breast | 18/29 62% | 0/13 0% | 0.001 |
| Mesa-Tejada 1978 [2] | US | Invasive | Normal breast | 51/131 39% | 0/18 0% | 0.001 |
| Wang 1995 [3] | US | Invasive | Normal breast | 121/314  38.5% | 2/107  2% | 0.001 |
| Etkind 2000 [4] | US | Invasive | Normal breast | 27/73 37% | 0/35 0% | 0.001 |
| Melana 2001 [5] | US | Invasive | Adjacent normal breast | 32/106 30% | 1/106 1% | 0.001 |
| Melana 2002 [6] | Argentina | Invasive | Normal breast | 23/74 31% | 1/10 10% | 0.001 |
| Ford 2003 [7] | Australia | DCIS  Invasive | Benign | DCIS 5/19 26%  IDC 14/26  54% | 2/111 2% | 0.001 |
| Ford 2004 [8] | Australia | Invasive  DCIS | Normal breast | IDC 45/144 31% | 0/20 0% | 0.001 |
| Zammarchi 2006 [9] | Italy | Invasive | Adjacent normal breast | 13/43 30% | 1/8 12.5% | 0.001 |
| Hachana 2008 [10] | Tunisia | Invasive | Adjacent normal breast | 17/122 14% | 0/122 0% | 0.001 |
| Lawson 2010 [11] | Australia | Invasive  DCIS | Normal  cosmetic | 33/74  45% | 0/29 0% | 0.001 |
| Mazzanti 2011[12] | Italy | DCIS  Invasive | Normal cosmetic | DCIS 40/49 82%  IDC 7/20 35% | 0/20 0% | 0.001 |
| Glenn 2012[13] | Australia | Invasive | Normal Human milk cells. | 39/50 78% | 13/40 33% | 0.003 |
| Slaoui 2014 [14] | Morocco | Invasive | Adjacent normal breast | 24/57 42% | 6/18 33% | 0.001 |
| Naushad 2014 [15] | Pakistan | Invasive | Normal | 83/250 29% | 0/15 0% | 0.001 |
| Cedro 2014 [16] | Mexico | Invasive | Adjacent normal breast | 57/458 12% | 72/458 16% | 0.187 ns |
| Reza 2015 [17] | Iran | Invasive  DCIS | Adjacent normal breast | 12/100 12% | 0/100 12% | 0.001 |
| Shariatpanahi 2017 [18] | Iran | Invasive | Benign | 19/59 32% | 3/59 5% | 0.001 |
| Al Dossary 2018 [19] | Saudi Arabia | Invasive | Normal benign | 6/101 5.9% | 0/51 0% | 0.05 |
| Seo 2019 [20] | Korea | Invasive | Adjacent normal breast | 12/128 9.4% | 0/128 0% | 0.01 |
| Al Hamad 2020 [21] | Jordan | Invasive | Normal breast | 11/100 11% | 0/20 0% | 0.016 |
| Pereira 2020 [22] | Brazil | Invasive | Adjacent normal breast | 41/217 18.9% | 30/196 15.3% | 0.417 ns |

**Supplementary Table 1. Identification of MMTV sequences in breast cancer (Case control studies)**

DCIS – ductal carcinoma in situ; IDC – invasive ductal carcinoma; ns = not significant at 0.05 level

**References MMTV**

1. Axel R, Schlom J, Spiegelman S. Presence in Human Breast Cancer of RNA

homologous to Mouse Mammary Tumour Virus RNA. Nature 1972;235:32-36.

2. Mesa-Tejada R, Keydar I, Ramanarayanan M, Ohno T, Fenoglio C, Spiegelman S. Detection in human breast carcinomas of an antigen immunologically related to a group-specific antigen of mouse mammary tumor virus. Proc Natl Acad Sci USA 1978;75:1529–1533.

3. Wang Y, Holland JF, Bleiweiss IJ, Melana S, Liu X, Pelisson L, Cantarella A, Stellrecht K, Mani S, Pogo BGT. Detection of mammary tumor virus env gene-like sequences in human breast cancer. Cancer Res. 1995;55:5173–5179.

4. Etkind, P., Du, J., Khan, A., Pillitteri, J. & Wiernik, P. H. Mouse mammary tumor

virus-like ENV gene sequences in human breast tumors and in a lymphoma of a

breast cancer patient. Clin Cancer Res. 2000;6:1273–1278.

5. Melana S, Holland, J. F, Pogo B G. Search for mouse mammary tumor virus like

env sequences in cancer and normal breast from the same individuals. Clin Cancer Res. 2001;7:283–284.

6. Melana SM, Picconi MA, Rossi C, Mural J, Alonio LV, Teyssié A, Holland JF, Pogo BG. Detection of murine mammary tumor virus (MMTV) env gene like sequences in breast cancer from Argentine patients. Med (B Aires) 2002;62:323–327. (Spanish).

7. Ford C E, Tran D D, Deng Y M, Rawlinson W D, Lawson J S. Mouse mammary tumour like virus prevalence in breast tumours of Australian and Vietnamese women. Clin Cancer Res. 2003;9:1118–1120.

8. Ford C E, Faedo M, Crouch R, Lawson J S, Rawlinson, W. D. Progression from

normal breast pathology to breast cancer is associated with increasing prevalence of mouse mammary tumor virus-like sequences in men and women. Cancer Res 2004;64:4755–4759.

9. [Zammarchi F](https://www.ncbi.nlm.nih.gov/pubmed/?term=Zammarchi%20F%5BAuthor%5D&cauthor=true&cauthor_uid=16710841), [Pistello M](https://www.ncbi.nlm.nih.gov/pubmed/?term=Pistello%20M%5BAuthor%5D&cauthor=true&cauthor_uid=16710841), [Piersigilli A](https://www.ncbi.nlm.nih.gov/pubmed/?term=Piersigilli%20A%5BAuthor%5D&cauthor=true&cauthor_uid=16710841), [Murr R](https://www.ncbi.nlm.nih.gov/pubmed/?term=Murr%20R%5BAuthor%5D&cauthor=true&cauthor_uid=16710841), [Di Cristofano C](https://www.ncbi.nlm.nih.gov/pubmed/?term=Di%20Cristofano%20C%5BAuthor%5D&cauthor=true&cauthor_uid=16710841), [Naccarato AG](https://www.ncbi.nlm.nih.gov/pubmed/?term=Naccarato%20AG%5BAuthor%5D&cauthor=true&cauthor_uid=16710841), [Bevilacqua G](https://www.ncbi.nlm.nih.gov/pubmed/?term=Bevilacqua%20G%5BAuthor%5D&cauthor=true&cauthor_uid=16710841). MMTV-like sequences in human breast cancer: a fluorescent PCR/laser microdissection approach. J Pathol 2006;209:436.

10. Hachana M, Trimeche M, Ziadi S, Amara K, Gaddas N, Mokni M, Korbi S. Prevalence and characteristics of the MMTV-like associated breast carcinomas in Tunisia. Cancer Lett 2008;271:222–230.

11. Lawson JS, Glenn WK, Salmons B, Ye Y, Heng B, Moody P, Johal H, Rawlinson WD, Delprado W, Lutze-Mann L, Whitaker NJ. [Mouse mammary tumor virus-like sequences in human breast cancer.](https://www.ncbi.nlm.nih.gov/pubmed/20388779) Cancer Res 2010;70:3576-3585.

12. [Mazzanti CM](https://www.ncbi.nlm.nih.gov/pubmed/?term=Mazzanti%20CM%5BAuthor%5D&cauthor=true&cauthor_uid=21854742), [Al Hamad M](https://www.ncbi.nlm.nih.gov/pubmed/?term=Al%20Hamad%20M%5BAuthor%5D&cauthor=true&cauthor_uid=21854742), [Fanelli G](https://www.ncbi.nlm.nih.gov/pubmed/?term=Fanelli%20G%5BAuthor%5D&cauthor=true&cauthor_uid=21854742), [Scatena C](https://www.ncbi.nlm.nih.gov/pubmed/?term=Scatena%20C%5BAuthor%5D&cauthor=true&cauthor_uid=21854742), [Zammarchi F](https://www.ncbi.nlm.nih.gov/pubmed/?term=Zammarchi%20F%5BAuthor%5D&cauthor=true&cauthor_uid=21854742), [Zavaglia K](https://www.ncbi.nlm.nih.gov/pubmed/?term=Zavaglia%20K%5BAuthor%5D&cauthor=true&cauthor_uid=21854742), [Lessi F](https://www.ncbi.nlm.nih.gov/pubmed/?term=Lessi%20F%5BAuthor%5D&cauthor=true&cauthor_uid=21854742), [Pistello M](https://www.ncbi.nlm.nih.gov/pubmed/?term=Pistello%20M%5BAuthor%5D&cauthor=true&cauthor_uid=21854742), [Naccarato AG](https://www.ncbi.nlm.nih.gov/pubmed/?term=Naccarato%20AG%5BAuthor%5D&cauthor=true&cauthor_uid=21854742), [Bevilacqua G](https://www.ncbi.nlm.nih.gov/pubmed/?term=Bevilacqua%20G%5BAuthor%5D&cauthor=true&cauthor_uid=21854742). A mouse mammary tumor virus env-like exogenous sequence is strictly related to progression of human sporadic breast carcinoma. [Am J Pathol.](https://www.ncbi.nlm.nih.gov/pubmed/?term=mazzanti+2011++MMTV) 2011;179:2083-2090.

13. Glenn WK, Heng B, Delprado W, Iacopetta B, Whitaker NJ, Lawson JS. Epstein-Barr virus, human papillomavirus and mouse mammary tumour virus as multiple viruses in breast cancer. PLoS ONE 2012;7:e48788 (2012).

14. Slaoui M, Mzibri ME, Razine R, Qmichou Z, Attaleb M, Amrani M. Detection of MMTV-Like sequences in Moroccan breast cancer cases. Infect Agent Cancer 2014;9:37.

15. [Naushad W](https://www.ncbi.nlm.nih.gov/pubmed/?term=Naushad%20W%5BAuthor%5D&cauthor=true&cauthor_uid=24839004), [Bin Rahat T](https://www.ncbi.nlm.nih.gov/pubmed/?term=Bin%20Rahat%20T%5BAuthor%5D&cauthor=true&cauthor_uid=24839004), [Gomez MK](https://www.ncbi.nlm.nih.gov/pubmed/?term=Gomez%20MK%5BAuthor%5D&cauthor=true&cauthor_uid=24839004), [Ashiq MT](https://www.ncbi.nlm.nih.gov/pubmed/?term=Ashiq%20MT%5BAuthor%5D&cauthor=true&cauthor_uid=24839004), [Younas M](https://www.ncbi.nlm.nih.gov/pubmed/?term=Younas%20M%5BAuthor%5D&cauthor=true&cauthor_uid=24839004), [Sadia H](https://www.ncbi.nlm.nih.gov/pubmed/?term=Sadia%20H%5BAuthor%5D&cauthor=true&cauthor_uid=24839004). Detection and identification of mouse mammary tumor virus like DNA sequences in blood and breast tissues of breast cancer patients. Tumour Biol 2014;35:8077–8086.

16. Cedro-Tanda A, Córdova-Solis A, Juárez-Cedillo T, Pina-Jiménez E, Hernández-Caballero ME, Moctezuma-Meza C et al. [Prevalence of HMTV in breast carcinomas and unaffected tissue from Mexican women.](https://www.ncbi.nlm.nih.gov/pubmed/25495285) BMC Cancer 2014;14:942.

17. Reza MA, Reza MH, Mahdiyeh L, Mehdi F, Hamid ZN. Evaluation Frequency of Merkel Cell Polyoma, Epstein-Barr and Mouse Mammary Tumor Viruses in Patients with Breast Cancer in Kerman, Southeast of Iran. Asian Pac J Cancer Prev, 2015;16:7351-7357.

18. Shariatpanahi, S., Farahani, N., Salehi, A. R, Salehi, R. High prevalence of mouse mammary tumor virus-like gene sequences in breast cancer samples of Iranian

women. Nucleosides Nucleotides Nucleic Acids 2017;36:621–630.

19. Al Dossary R, Alkharsah K R, Kussaibi H. Prevalence of Mouse Mammary

Tumor Virus (MMTV)-like sequences in human breast cancer tissues and adjacent

normal breast tissues in Saudi Arabia. BMC Cancer 2018;18:170.

20. Seo I, Cho JH, Lee MH, Park WJ, Kwon SY, Lee JH. [Clinical and Prognostic Value of Human Mammary Tumor Virus in Korean Patients with Breast Carcinoma.](https://www.ncbi.nlm.nih.gov/pubmed/31028060) Annals Clin Lab Science. 2019;49:171-174.

21. Al Hamad M, Matalka I, Al Zoubi MS, Armogida I, Khasawneh R, Al-Husaini M, Sughayer M, Jaradat S, Al-Nasser AD, Mazzanti CM. [Human mammary tumor virus, human papilloma virus, and Epstein-Barr Virus infection are associated with sporadic breast cancer metastasis.](https://pubmed.ncbi.nlm.nih.gov/33281452/) Breast Cancer (Auckl). 2020;14:1178223420976388.

[22. Pereira](https://pubmed.ncbi.nlm.nih.gov/?sort=pubdate&size=50&term=de+Sousa+Pereira+N&cauthor_id=33352945) N de S ,  [Vitiello](https://pubmed.ncbi.nlm.nih.gov/?sort=pubdate&size=50&term=Akelinghton+Freire+Vitiello+G&cauthor_id=33352945) GAF ,  [Banin-Hirata](https://pubmed.ncbi.nlm.nih.gov/?sort=pubdate&size=50&term=Karina+Banin-Hirata+B&cauthor_id=33352945) BK ,  [Fernandes](https://pubmed.ncbi.nlm.nih.gov/?sort=pubdate&size=50&term=Scantamburlo+Alves+Fernandes+G&cauthor_id=33352945) GSA ,  [Salles](https://pubmed.ncbi.nlm.nih.gov/?sort=pubdate&size=50&term=Jos%C3%A9+Spar%C3%A7a+Salles+M&cauthor_id=33352945) MJS ,  [Amarante](https://pubmed.ncbi.nlm.nih.gov/?sort=pubdate&size=50&term=Karine+Amarante+M&cauthor_id=33352945) MK ,  [Watanabe](https://pubmed.ncbi.nlm.nih.gov/?sort=pubdate&size=50&term=Angelica+Ehara+Watanabe+M&cauthor_id=33352945) MAE. Mouse Mammary Tumor Virus (MMTV)-Like *env* Sequence in Brazilian Breast Cancer Samples: Implications in Clinicopathological Parameters in Molecular Subtypes. Int J Environ Res Public Health. 2020;17(24):9496.

| Study | Country | HPV Breast cancer | HPV Non cancer breast controls | Main HPV types | P value for difference cancer/ control |
| --- | --- | --- | --- | --- | --- |
| Yu 2000[1] | Japan / China | 18/52 35% | 0/15 0% | 18,33 | 0.001 |
| Ren 2003[2] | China | 45/80 56% | 2/30 7% | 16,18 | 0.002 |
| Damin 2004[3] | Brazil | 25/101 25% | 0/41 0% | 16,18 | 0.001 |
| Tsai 2007 [4] | Taiwan | 8/62 13% | 2/32 6% |  | 0.004 |
| Choi 2007 [5] | Korea | 8/123 7% | 0/31 0% | 16, 18,58 | 0.001 |
| Gumus 2006 [6] | Turkey | 37/50 74% | 16/50 32% | 18,33 | 0.162 ns |
| Fan 2008[7] | China | 23/52 44% | 1/16 6% | 16 | 0.001 |
| He 2009 [8] | China | 24/40 60% | 1/20 5% | 16 | 0.001 |
| De Leon 2009 [9] | Mexico | 15/41 37% | 0/43 0% | 16,18 | 0.001 |
| Mendizabal 2009 [10] | Mexico | 3/67 4% | 0/40 0% | 16,18,33 | 0.157ns |
| Heng 2009 [11] | Australia | 8/26 31% | 3/28 11% | 16,18 | 0.611ns |
| Mou 2011 [12] | China | 4/62 6% | 0/46 0% | 16,18 | 0.025 |
| Sigaroodi 2012 [13] | Iran | 15/58 26% | 1/41 2% | 16,18 | 0.002 |
| Frega 2012[14] | Italy | 9/31 29% | 0/12 0% | 16,18 | 0.005 |
| Divani 2012[15] | Greece | 6/35 17% | 0/35 0% | 16,18 | 0.025 |
| Glenn 2012 [16] | Australia | 25/50 50% | 8/40 20% | 16,18 | 0.006 |
| Liang 2013 [17] | China | 48/224 21% | 6/37 16% | 16,18,33,58 | 0.001 |
| Ahangar2014[18] | Iran | 22/65 34% | 0/65 0% | 16 | 0.001 |
| Ali 2014 [19] | Iraq | 60/129 47% | 3/41 7% | 16,18,33 | 0.001 |
| Hong 2014 [20] | China | 23/45 51% | 1/20 5% | 16,18 | 0.001 |
| Manzouri 2014 [21] | Iran | 10/55 18% | 7/51 14% | 16 | 0.083 ns |
| Fu 2015 [22] | China | 25/169 15% | 1/83 1% | 58 | 0.001 |
| Li 2015 [23] | China | 3/187 2% | 0/92 0% | 6,16,18 | 0.157 ns |
| Gannon 2015[24] | Australia | 13/78 17% | 1/10 10% |  | 0.002 |
| Lawson 2015 [25] | Australia | 27/40 66% | 6/21 29% | 16,18,58 | 0.001 |
| Doosti 2016[26] | Iran | 20/87 23% | 0/84 0% | 16,18 | 0.001 |
| Wang 2016 [27] | China | 52/146 36% | 3/83 3.6% | 16,18,58 | 0.001 |
| Zhang 2016 [28] | China | 34/325 11% | 4/100 4% | 16,18 | 0.001 |
| Delgardo 2017 [29] | Spain | 130/251 52% | 49/186 26% | 16 | 0.001 |
| Ladera 2017 [30] | Venezuela | 14/22 64% | 1/22 4.5% | 16,18,52,56 | 0.001 |
| Naushad 2017 [31] | Pakistan | 45/250 18% | 0/15 0% |  | 0.001 |
| Islam 2017 [32] | India | 203/313 65% | 2/21 10% | 16,18,33 | 0.001 |
| Salman 2017 [33] | United Kingdom | 35/74 47% | 11/36 18% | 16,18,35,45,59 | 0.001 |
| Malekpour 2018 [34] | Iran | 8/98 8% | 0/40 0% | 16,18 | 0.008 |
| ElAmrani 2018 [35] | Morocco | 19/76 25% | 1/12 8% | 51,52,58 | 0.001 |
| Cavalcante 2018 [36] | Brazil | 51/103 50% | 15/95 16% | 6,11,18,31 | 0.001 |
| Khodabandehlou 2019 [37] | Iran | 35/72 48.6% | 5/3116.1% | 18 | 0.003 |
| Al Hamad 2020 [38] | Jordan | 21/100 21% | 0/20 0% | 16, 18 | 0.007 |
| Mofrad 2021 [39] | Iran | 7/59 12% | 0/11 0% | 18 | 0.004 |
| El-Seik 2021 [40] | Egypt | 16/72 22.2% | 0/15 0% | 16,18 | 0.003 |

**Supplementary Table 2. identification of high risk for cancer human papilloma virus in breast cancers and controls (case control studies).**

The prevalence of high risk HPV is consistently higher in all studies of breast cancers as compared to controls. The difference is statistically significant for 22 of 25 studies.

ns = not significant at 0.05 level

**References**

1. Yu Y, Morimoto T, Sasa M, Okazaki K, Harada Y, Fujiwara T et al. [Human papillomavirus type 33 DNA in breast cancer in Chinese.](https://www.ncbi.nlm.nih.gov/pubmed/11029768) Breast Cancer. 2000;7:33-36.

2. Ren Z, Huang J, Shi Z, et al. Detection of human papillomavirus types 16 and 18 infection in breast cancer tissues by Primed in situ labeling. Zhongguo Zhongliu Linchuang 30:243-246 (2003). (Data from Ren et al 2019)

3. [Damin AP](https://www.ncbi.nlm.nih.gov/pubmed/?term=Damin%20AP%5BAuthor%5D&cauthor=true&cauthor_uid=14999143), [Karam R](https://www.ncbi.nlm.nih.gov/pubmed/?term=Karam%20R%5BAuthor%5D&cauthor=true&cauthor_uid=14999143), [Zettler CG](https://www.ncbi.nlm.nih.gov/pubmed/?term=Zettler%20CG%5BAuthor%5D&cauthor=true&cauthor_uid=14999143), [Caleffi M](https://www.ncbi.nlm.nih.gov/pubmed/?term=Caleffi%20M%5BAuthor%5D&cauthor=true&cauthor_uid=14999143), [Alexandre CO](https://www.ncbi.nlm.nih.gov/pubmed/?term=Alexandre%20CO%5BAuthor%5D&cauthor=true&cauthor_uid=14999143). Evidence for an association of human papillomavirus and breast carcinomas. [Breast Cancer Res Treat.](https://www.ncbi.nlm.nih.gov/pubmed/?term=damin+2004+hpv) 2004;84:131-137.

4. Tsai JH, Hsu CS, Tsai CH, Su JM, Liu YT, Cheng MH et al. [Relationship between viral factors, axillary lymph node status and survival in breast cancer.](https://www.ncbi.nlm.nih.gov/pubmed/16865407) J Cancer Res Clin Oncol. 2007;133:13-21.

5. Choi YL, Cho EY, Kim JH, Nam SJ, Oh YL, Song SY et al. [Detection of human papillomavirus DNA by DNA chip in breast carcinomas of Korean women.](https://www.ncbi.nlm.nih.gov/pubmed/18391549) Tumour Biol. 2007; 28:327-332.

6. [Gumus M](https://www.ncbi.nlm.nih.gov/pubmed/?term=Gumus%20M%5BAuthor%5D&cauthor=true&cauthor_uid=17310842), [Yumuk PF](https://www.ncbi.nlm.nih.gov/pubmed/?term=Yumuk%20PF%5BAuthor%5D&cauthor=true&cauthor_uid=17310842), [Salepci T](https://www.ncbi.nlm.nih.gov/pubmed/?term=Salepci%20T%5BAuthor%5D&cauthor=true&cauthor_uid=17310842), [Aliustaoglu M](https://www.ncbi.nlm.nih.gov/pubmed/?term=Aliustaoglu%20M%5BAuthor%5D&cauthor=true&cauthor_uid=17310842), [Dane F](https://www.ncbi.nlm.nih.gov/pubmed/?term=Dane%20F%5BAuthor%5D&cauthor=true&cauthor_uid=17310842), [Ekenel M](https://www.ncbi.nlm.nih.gov/pubmed/?term=Ekenel%20M%5BAuthor%5D&cauthor=true&cauthor_uid=17310842) et al. HPV DNA frequency and subset analysis in human breast cancer patients' normal and tumoral tissue samples. [J Exp Clin Cancer Res.](https://www.ncbi.nlm.nih.gov/pubmed/?term=gumus+2006++hpv) 2006;25:515-521.

7. Fan CL, Zhou JH, Hu CY. Expression of human papillomavirus in mammary carcinoma and its possible mechanism in carcinogenesis. Virologica Sinica 2008;23:226-231.

8. He Q, Zhang SQ, Chu YL, Jia XL, Wang XL. The correlations between HPV16 infection and expressions of c-erbB-2 and bcl-2 in breast carcinoma. Mol Biol Rep. 2009;36:807–812.

9. de León DC, Montiel DP, Nemcova J, Mykyskova I, Turcios E, Villavicencio V, et al. Human papillomavirus (HPV) in breast tumors: prevalence in a group of Mexican patients. BMC Cancer. 2009;9:26.

10. [Mendizabal-Ruiz AP](https://www.ncbi.nlm.nih.gov/pubmed/?term=Mendizabal-Ruiz%20AP%5BAuthor%5D&cauthor=true&cauthor_uid=18373273), [Morales JA](https://www.ncbi.nlm.nih.gov/pubmed/?term=Morales%20JA%5BAuthor%5D&cauthor=true&cauthor_uid=18373273), [Ramírez-Jirano LJ](https://www.ncbi.nlm.nih.gov/pubmed/?term=Ram%C3%ADrez-Jirano%20LJ%5BAuthor%5D&cauthor=true&cauthor_uid=18373273), [Padilla-Rosas M](https://www.ncbi.nlm.nih.gov/pubmed/?term=Padilla-Rosas%20M%5BAuthor%5D&cauthor=true&cauthor_uid=18373273), [Morán-Moguel MC](https://www.ncbi.nlm.nih.gov/pubmed/?term=Mor%C3%A1n-Moguel%20MC%5BAuthor%5D&cauthor=true&cauthor_uid=18373273), [Montoya-Fuentes H](https://www.ncbi.nlm.nih.gov/pubmed/?term=Montoya-Fuentes%20H%5BAuthor%5D&cauthor=true&cauthor_uid=18373273). Low frequency of human papillomavirus DNA in breast cancer tissue. Breast Cancer Res Treat 2009;114:189-194 .

11. Heng B, Glenn WK, Ye Y, Tran B, Delprado W, Lutze-Mann L, et al. Human papilloma virus is associated with breast cancer. Br J Cancer. 2009;101:1345–1350.

12. Mou X, Chen L, Liu F, Shen Y, Wang H, Li Y, et al. Low prevalence of human papillomavirus (HPV) in Chinese patients with breast cancer. J Int Med Res. 2011;39:1636–1644.

13. Sigaroodi A, Nadji SA, Naghshvar F, Nategh R, Emami H, Velayati AA. Human papillomavirus is associated with breast cancer in the north part of Iran. Scientific World J. 2012:837191.

14. Frega A, Lorenzon L, Bononi M, De Cesare A, Ciardi A, Lombardi D et al. [Evaluation of E6 and E7 mRNA expression in HPV DNA positive breast cancer.](https://www.ncbi.nlm.nih.gov/pubmed/22611956) Eur J Gynaecol Oncol. 2012;33:164-167.

15. Divani SN, Giovani AM. Detection of human papillomavirus DNA in fine needle aspirates of women with breast cancer. Arch Oncol. 2012;20:12-14.

16. Glenn WK, Heng B, Delprado W, Iacopetta B, Whitaker NJ, Lawson JS. [Epstein-Barr virus, human papillomavirus and mouse mammary tumour virus as multiple viruses in breast cancer.](https://www.ncbi.nlm.nih.gov/pubmed/23183846) PLoS One.2012;7:e48788.

17. Liang W, Wang J, Wang C, Lv Y, Gao H, Zhang K, et al. Detection of high-risk human papillomaviruses in fresh breast cancer samples using the hybrid capture 2 assay. J Med Virol. 2013;85:2087–2092.

18. Ahangar-Oskouee M, Shahmahmoodi S, Jalilvand S, Mahmoodi M, Ziaee AA, Esmaeili HA, et al. No detection of ‘high-risk’ human papillomaviruses in a group of Iranian women with breast cancer. Asian Pac J Cancer Prev.2014;15:4061–4065.

19. Ali SH, Al-Alwan NA, Al-Alwany SH. [Detection and genotyping of human papillomavirus in breast cancer tissues from Iraqi patients.](https://www.ncbi.nlm.nih.gov/pubmed/24960513) East Mediterr Health J. 2014;20:372-377.

20. Hong L, Tang S. Does HPV 16/18 infection affect p53 expression in invasive ductal carcinoma? An experimental study. Pak J Med Sci 2014;30:789-792.

21. Manzouri L, Salehi R, Shariatpanahi S, Rezaie P. Prevalence of human papilloma virus among women with breast cancer since 2005–2009 in Isfahan. Adv Biomed Res.2014;3:75.

22. Fu L, Wang D, Shah W, Wang Y, Zhang G, He J. Association of human papillomavirus type 58 with breast cancer in Shaanxi province of China. J Med Virol. 2015;87:1034–1040.

23. Li J, Ding J, Zhai K. [Detection of Human Papillomavirus DNA in Patients with Breast Tumor in China.](https://www.ncbi.nlm.nih.gov/pubmed/26295705) PLoS One. 2015;10:e0136050.

24. Gannon OM, Antonsson A, Milevskiy M, Brown MA, Saunders NA, Bennett IC. [No association between HPV positive breast cancer and expression of human papilloma viral transcripts.](https://www.ncbi.nlm.nih.gov/pubmed/26658849) Sci Rep. 2015;5:18081.

25. Lawson JS, Glenn WK, Salyakina D, Delprado W, Clay R, Antonsson A, Heng B, Miyauchi S, Tran DD, Ngan CC, Lutze-Mann L, Whitaker NJ. [Human Papilloma Viruses and Breast Cancer.](https://www.ncbi.nlm.nih.gov/pubmed/26734565) Front Oncol 2015;5:277.

26. [Doosti M](https://www.ncbi.nlm.nih.gov/pubmed/?term=Doosti%20M%5BAuthor%5D&cauthor=true&cauthor_uid=27797244), [Bakhshesh M](https://www.ncbi.nlm.nih.gov/pubmed/?term=Bakhshesh%20M%5BAuthor%5D&cauthor=true&cauthor_uid=27797244), [Zahir ST](https://www.ncbi.nlm.nih.gov/pubmed/?term=Zahir%20ST%5BAuthor%5D&cauthor=true&cauthor_uid=27797244), [Shayestehpour M](https://www.ncbi.nlm.nih.gov/pubmed/?term=Shayestehpour%20M%5BAuthor%5D&cauthor=true&cauthor_uid=27797244), [Karimi-Zarchi M](https://www.ncbi.nlm.nih.gov/pubmed/?term=Karimi-Zarchi%20M%5BAuthor%5D&cauthor=true&cauthor_uid=27797244). Lack of Evidence for a Relationship between High Risk Human Papillomaviruses and Breast Cancer in Iranian Patients. Asian Pac J Cancer Prev 2016;17:4357-4361.

27. Wang D, Fu L, Shah W, Zhang J, Yan Y, Ge X, He J, Wang Y, Li X. [Presence of high risk HPV DNA but indolent transcription of E6/E7 oncogenes in invasive ductal carcinoma of breast.](https://www.ncbi.nlm.nih.gov/pubmed/27688086) Pathol Res Pract 2016;212:1151-1156.

28. Zhang N, Ma ZP, Wang J, Bai HL, Li YX, Sun Q et al. Human papillomavirus infection correlates with inflammatory Stat3 signaling activity and IL-17 expression in patients with breast cancer. Am J Transl Res 2016;8:3214-3226.29.

29. [Delgado-García S](https://www.ncbi.nlm.nih.gov/pubmed/?term=Delgado-Garc%C3%ADa%20S%5BAuthor%5D&cauthor=true&cauthor_uid=28482874), [Martínez-Escoriza JC](https://www.ncbi.nlm.nih.gov/pubmed/?term=Mart%C3%ADnez-Escoriza%20JC%5BAuthor%5D&cauthor=true&cauthor_uid=28482874), [Alba A](https://www.ncbi.nlm.nih.gov/pubmed/?term=Alba%20A%5BAuthor%5D&cauthor=true&cauthor_uid=28482874), [Martín-Bayón TA](https://www.ncbi.nlm.nih.gov/pubmed/?term=Mart%C3%ADn-Bay%C3%B3n%20TA%5BAuthor%5D&cauthor=true&cauthor_uid=28482874), [Ballester-Galiana H](https://www.ncbi.nlm.nih.gov/pubmed/?term=Ballester-Galiana%20H%5BAuthor%5D&cauthor=true&cauthor_uid=28482874), [Peiró G](https://www.ncbi.nlm.nih.gov/pubmed/?term=Peir%C3%B3%20G%5BAuthor%5D&cauthor=true&cauthor_uid=28482874) et al. Presence of human papillomavirus DNA in breast cancer: a Spanish case-control study. [BMC Cancer.](https://www.ncbi.nlm.nih.gov/pubmed/?term=Delgado-Garc%C3%ADa+2017+hpv) 2017;17:320.

# 30. Ladera M, Fernandes A, López M, Pesci-Feltri A, Ávila M, Correnti M. Presence of human papillomavirus and Epstein-Barr virus in breast cancer biopsies as potential risk factors. Gaceta Mexicana de Oncologia 2017;16:107-112.

# 31. [Naushad W](https://www.ncbi.nlm.nih.gov/pubmed/?term=Naushad%20W%5BAuthor%5D&cauthor=true&cauthor_uid=28705719), [Surriya O](https://www.ncbi.nlm.nih.gov/pubmed/?term=Surriya%20O%5BAuthor%5D&cauthor=true&cauthor_uid=28705719), [Sadia H](https://www.ncbi.nlm.nih.gov/pubmed/?term=Sadia%20H%5BAuthor%5D&cauthor=true&cauthor_uid=28705719). Prevalence of EBV, HPV and MMTV in Pakistani breast cancer patients: A possible etiological role of viruses in breast cancer. [Infect Genet Evol.](https://www.ncbi.nlm.nih.gov/pubmed/?term=naushad++2017+hpv+breast) 2017;54:230-237.

32. Islam S, Dasgupta H, Roychowdhury A, Bhattacharya R, Mukherjee N, Roy A, et al. Study of association and molecular analysis of human papillomavirus in breast cancer of Indian patients: Clinical and prognostic implication. PLoS One 2017;12: e0172760.

33. [Salman NA](https://www.ncbi.nlm.nih.gov/pubmed/?term=Salman%20NA%5BAuthor%5D&cauthor=true&cauthor_uid=28240743), [Davies G](https://www.ncbi.nlm.nih.gov/pubmed/?term=Davies%20G%5BAuthor%5D&cauthor=true&cauthor_uid=28240743), [Majidy F](https://www.ncbi.nlm.nih.gov/pubmed/?term=Majidy%20F%5BAuthor%5D&cauthor=true&cauthor_uid=28240743), [Shakir F](https://www.ncbi.nlm.nih.gov/pubmed/?term=Shakir%20F%5BAuthor%5D&cauthor=true&cauthor_uid=28240743), [Akinrinade H](https://www.ncbi.nlm.nih.gov/pubmed/?term=Akinrinade%20H%5BAuthor%5D&cauthor=true&cauthor_uid=28240743), [Perumal D](https://www.ncbi.nlm.nih.gov/pubmed/?term=Perumal%20D%5BAuthor%5D&cauthor=true&cauthor_uid=28240743) et al. Association of High Risk Human Papillomavirus and Breast cancer: A UK based Study. [Sci Rep.](https://www.ncbi.nlm.nih.gov/pubmed/?term=salman+2017++hpv+breast) 2017;7:43591.

34. [Malekpour AR](https://www.ncbi.nlm.nih.gov/pubmed/?term=Malekpour%20Afshar%20R%5BAuthor%5D&cauthor=true&cauthor_uid=29373913), [Deldar Z](https://www.ncbi.nlm.nih.gov/pubmed/?term=Deldar%20Z%5BAuthor%5D&cauthor=true&cauthor_uid=29373913), [Mollaei HR](https://www.ncbi.nlm.nih.gov/pubmed/?term=Mollaei%20HR%5BAuthor%5D&cauthor=true&cauthor_uid=29373913), [Arabzadeh SA](https://www.ncbi.nlm.nih.gov/pubmed/?term=Arabzadeh%20SA%5BAuthor%5D&cauthor=true&cauthor_uid=29373913), [Iranpour M](https://www.ncbi.nlm.nih.gov/pubmed/?term=Iranpour%20M%5BAuthor%5D&cauthor=true&cauthor_uid=29373913). Evaluation of HPV DNA positivity in colorectal cancer patients in Kerman, Southeast Iran. [Asian Pac J Cancer Prev.](https://www.ncbi.nlm.nih.gov/pubmed/?term=malekpour+2018+hpv)2018;19:193-198.

35. [ElAmrani A](https://www.ncbi.nlm.nih.gov/pubmed/?term=ElAmrani%20A%5BAuthor%5D&cauthor=true&cauthor_uid=29660489), [Gheit T](https://www.ncbi.nlm.nih.gov/pubmed/?term=Gheit%20T%5BAuthor%5D&cauthor=true&cauthor_uid=29660489), [Benhessou M](https://www.ncbi.nlm.nih.gov/pubmed/?term=Benhessou%20M%5BAuthor%5D&cauthor=true&cauthor_uid=29660489), [McKay-Chopin S](https://www.ncbi.nlm.nih.gov/pubmed/?term=McKay-Chopin%20S%5BAuthor%5D&cauthor=true&cauthor_uid=29660489), [Attaleb M](https://www.ncbi.nlm.nih.gov/pubmed/?term=Attaleb%20M%5BAuthor%5D&cauthor=true&cauthor_uid=29660489), [Sahraoui S](https://www.ncbi.nlm.nih.gov/pubmed/?term=Sahraoui%20S%5BAuthor%5D&cauthor=true&cauthor_uid=29660489) et al. Prevalence of mucosal and cutaneous human papillomavirus in Moroccan breast cancer. [Papillomavirus Res.](https://www.ncbi.nlm.nih.gov/pubmed/?term=elamrani+2018++hpv) 2018;5:150-155.

36. [Cavalcante JR](https://www.ncbi.nlm.nih.gov/pubmed/?term=Cavalcante%20JR%5BAuthor%5D&cauthor=true&cauthor_uid=30365827), [Pinheiro LGP](https://www.ncbi.nlm.nih.gov/pubmed/?term=Pinheiro%20LGP%5BAuthor%5D&cauthor=true&cauthor_uid=30365827), [Almeida PRC](https://www.ncbi.nlm.nih.gov/pubmed/?term=Almeida%20PRC%5BAuthor%5D&cauthor=true&cauthor_uid=30365827), [Ferreira MVP](https://www.ncbi.nlm.nih.gov/pubmed/?term=Ferreira%20MVP%5BAuthor%5D&cauthor=true&cauthor_uid=30365827), [Cruz GA](https://www.ncbi.nlm.nih.gov/pubmed/?term=Cruz%20GA%5BAuthor%5D&cauthor=true&cauthor_uid=30365827), [Campelo TA](https://www.ncbi.nlm.nih.gov/pubmed/?term=Campelo%20TA%5BAuthor%5D&cauthor=true&cauthor_uid=30365827) et al. Association of breast cancer with human papillomavirus (HPV) infection in Northeast Brazil: molecular evidence. [Clinics (Sao Paulo).](https://www.ncbi.nlm.nih.gov/pubmed/30365827) 2018;73:e465.

37. Khodabandehlou N, Mostafaei S, Etemadi A, Ghasemi A, Payandeh M, Hadifar S et al. [Human papilloma virus and breast cancer: the role of inflammation and viral expressed proteins.](https://www.ncbi.nlm.nih.gov/pubmed/30642295) BMC Cancer. 2019;19:61.

38. Al Hamad M, Matalka I, Al Zoubi MS, Armogida I, Khasawneh R, Al-Husaini M, Sughayer M, Jaradat S, Al-Nasser AD, Mazzanti CM. [Human mammary tumor virus, human papilloma virus, and Epstein-Barr Virus infection are associated with sporadic breast cancer metastasis.](https://pubmed.ncbi.nlm.nih.gov/33281452/) Breast Cancer (Auckl). 2020;14:1178223420976388.

### 39. Mofrad MG, Sadigh ZA, Ainechi S, Faghihloo E. [Detection of human papillomavirus genotypes, herpes simplex, varicella zoster and cytomegalovirus in breast cancer patients](https://virologyj.biomedcentral.com/articles/10.1186/s12985-021-01498-z) Virology J 2021;18:25

40. El-Sheik N ,  [Mousa](https://pubmed.ncbi.nlm.nih.gov/?sort=pubdate&size=50&term=Mousa+NO&cauthor_id=33716506) NO , [Tawfeik](https://pubmed.ncbi.nlm.nih.gov/?sort=pubdate&size=50&term=Tawfeik+AM&cauthor_id=33716506) AM , [Saleh](https://pubmed.ncbi.nlm.nih.gov/?sort=pubdate&size=50&term=Saleh+AM&cauthor_id=33716506) AM , [Elshikh](https://pubmed.ncbi.nlm.nih.gov/?sort=pubdate&size=50&term=Elshikh+I&cauthor_id=33716506) I , [Deyab](https://pubmed.ncbi.nlm.nih.gov/?sort=pubdate&size=50&term=Deyab+M&cauthor_id=33716506) M et al.  Assessment of Human Papillomavirus Infection and Risk Factors in Egyptian Women With Breast Cancer. Breast Cancer (Auckl). 2021;15:1178223421996279.

| Study | Country | Identification method | EBV positive breast cancer | EBV positive breast controls | P value |
| --- | --- | --- | --- | --- | --- |
| Labreque 1995 [1] | United Kingdom | PCR, ISH | 19/91 21% | 0/21 0% | 0.001 |
| Luqmani 1995 [2] | United Kingdom | PCR, IHC | 15/28 54% | 0/12 0% | 0.001 |
| Bonnet 1999 [3] | France | PCR, IHC | 51/100 50% | 0/30 0% | 0.001 |
| Fina 2001 [4] | Algeria  Europe | PCR, ISH, microdissection | 162/509 32% | 0/10 0% | 0.001 |
| Grinstein 2002 [5] | United States | PCR, IHC | 14/33 42% | 3/26 12% | 0.039 |
| Preciado 2005 [6] | Argentina | PCR, IHC | 24/69 35% | 0/17 0% | 0.001 |
| Fawzy 2008 [7] | Egypt | PCR, IHC | 10/40 25% | 0/20 0% | 0.001 |
| Joshi 2009 [8] | India | IHC | 28/51 55% | 0/30 0% | 0.001 |
| Lorenzetti 2010 [9] | Argentina | PCR, ISH, IHC | 22/71 31% | 0/48 0% | 0.001 |
| Kadivar 2011[10] | Iran | PCR, IHC | 0/100 0% | 0/42 0% |  |
| Mazouni 2011 [11] | France | PCR  microdissection | 65/196 33% | 1/15 7% | 0.001 |
| Hachana 2011[12] | Tunisia | PCR, IHC | 33/90 | 0/123 | 0.001 |
| Glenn 2012 [13] | Australia | PCR, in situ PCR, IHC | 34/50 68% | 14/40 35% | 0.011 |
| Zekri 2012 [14] | Iraq | PCR, IHC, ISH | 32/90 35% | 0/20 0% | 0.001 |
| Khabaz 2013 [15] | Jordan | PCR, IHC | 24/92 26% | 3/49 6% | 0.001 |
| Yahia 2014 [16] | Sudan | PCR, ISH | 49/92 53% | 12/50 24% | 0.001 |
| Mohammadizadeh 2014 [17] | Iran | PCR, IHC | 6/74 8% | 0/80 0% | 0.001 |
| Richardson 2015 [18] | New Zealand | PCR | 24/70 34% | 9/70 13% | 0.253 ns |
| Ahmed 2016 [19] | Egypt | IHC | 11/10710% | 0/107 0% | 0.001 |
| El Naby 2017 [20] | Egypt | PCR, IHC | 10/42 24% | 6/42 14% | 0.689 ns |
| Fessahaye 2017 [21] | Eritrea | PCR, ISH, IHC | 40/144 28% | 4/33 12% | 0.003 |
| Pai 2018 [22] | India | ISH | 25/83 30% | 0/7 0% | 0.001 |
| Al Hamad 2020 [26] | Jordan | ISH | 24/100 24% | 0/20 0% | 0.007 |
| Alinezhad 2021 [27] | Iran | PCR | 9/80 11.2% | 0/80 0% | 0.009 |

**Supplementary Table 3. Case control studies Epstein Barr virus and breast cancer.**

PCR = polymerase chain reaction, IHC = immunohistochemistry, ISH = in situ hybridisation, ns = not significant at 0.05 level

**References**

1. Labrecque LG, Barnes DM, Fentiman IS, Griffin BE. Epstein-Barr virus in epithelial cell tumors: a breast cancer study. Cancer Res 1995:55:39–45.

2. Luqmani Y, Shousha S. [Presence of epstein-barr-virus in breast-carcinoma.](https://www.ncbi.nlm.nih.gov/pubmed/21556618) Int J Oncol 1995;6:899-903

3. Bonnet M, Guinebretiere JM, Kremmer E, Grunewald V, Benhamou E, Contesso G, et al. Detection of Epstein-Barr virus in invasive breast cancers. J Natl Cancer Inst 1999;91:1376–1381.

4.[Fina F](https://www.ncbi.nlm.nih.gov/pubmed/?term=Fina%20F%5BAuthor%5D&cauthor=true&cauthor_uid=11259092), [Romain S](https://www.ncbi.nlm.nih.gov/pubmed/?term=Romain%20S%5BAuthor%5D&cauthor=true&cauthor_uid=11259092), [Ouafik L](https://www.ncbi.nlm.nih.gov/pubmed/?term=Ouafik%20L%5BAuthor%5D&cauthor=true&cauthor_uid=11259092), [Palmari J](https://www.ncbi.nlm.nih.gov/pubmed/?term=Palmari%20J%5BAuthor%5D&cauthor=true&cauthor_uid=11259092), [Ben Ayed F](https://www.ncbi.nlm.nih.gov/pubmed/?term=Ben%20Ayed%20F%5BAuthor%5D&cauthor=true&cauthor_uid=11259092), [Benharkat S](https://www.ncbi.nlm.nih.gov/pubmed/?term=Benharkat%20S%5BAuthor%5D&cauthor=true&cauthor_uid=11259092), [Bonnier P](https://www.ncbi.nlm.nih.gov/pubmed/?term=Bonnier%20P%5BAuthor%5D&cauthor=true&cauthor_uid=11259092), [Spyratos F](https://www.ncbi.nlm.nih.gov/pubmed/?term=Spyratos%20F%5BAuthor%5D&cauthor=true&cauthor_uid=11259092), [Foekens JA](https://www.ncbi.nlm.nih.gov/pubmed/?term=Foekens%20JA%5BAuthor%5D&cauthor=true&cauthor_uid=11259092), [Rose C](https://www.ncbi.nlm.nih.gov/pubmed/?term=Rose%20C%5BAuthor%5D&cauthor=true&cauthor_uid=11259092), [Buisson M](https://www.ncbi.nlm.nih.gov/pubmed/?term=Buisson%20M%5BAuthor%5D&cauthor=true&cauthor_uid=11259092), [Gérard H](https://www.ncbi.nlm.nih.gov/pubmed/?term=G%C3%A9rard%20H%5BAuthor%5D&cauthor=true&cauthor_uid=11259092), [Reymond MO](https://www.ncbi.nlm.nih.gov/pubmed/?term=Reymond%20MO%5BAuthor%5D&cauthor=true&cauthor_uid=11259092), [Seigneurin JM](https://www.ncbi.nlm.nih.gov/pubmed/?term=Seigneurin%20JM%5BAuthor%5D&cauthor=true&cauthor_uid=11259092), [Martin PM](https://www.ncbi.nlm.nih.gov/pubmed/?term=Martin%20PM%5BAuthor%5D&cauthor=true&cauthor_uid=11259092). Frequency and genome load of Epstein-Barr virus in 509 breast cancers from different geographical areas. [Br J Cancer.](https://www.ncbi.nlm.nih.gov/pubmed/?term=fina+2001++EBV) 2001;84:783-790.

5. Grinstein S, Preciado MV, Gattuso P, Chabay PA, Warren WH, De Matteo E, et al. Demonstration of Epstein-Barr virus in carcinomas of various sites. Cancer Res 2002;62:4876–4878.

6. Preciado MV, Chabay PA, De Matteo EN, Gonzalez P, Grinstein S, Actis A, et al. Epstein-Barr virus in breast carcinoma in Argentina. Arch Pathol Lab Med 2005;129:377–381.

7. Fawzy S, Sallam M, Awad NM. Detection of Epstein-Barr virus in breast carcinoma in Egyptian women. Clin Biochem 2008;41:486–492.

8. Joshi D, Quadri M, Gangane N, Joshi R, Gangane N. Association of Epstein Barr virus infection (EBV) with breast cancer in rural Indian women. PLoS One 2009;4:e8180.

9. Lorenzetti MA, De Matteo E, Gass H, Martinez Vazquez P, Lara J, Gonzalez P, et al. Characterization of Epstein Barr virus latency pattern in Argentine breast carcinoma. PLoS One 2010;5:e13603.

10. Kadivar M, Monabati A, Joulaee A, Hosseini N. [Epstein-Barr virus and breast cancer: lack of evidence for an association in Iranian women.](https://www.ncbi.nlm.nih.gov/pubmed/21207256) Pathol Oncol Res 2011;17:489-492

11. Mazouni C, Fina F, Romain S, Ouafik L, Bonnier P, Brandone JM, Martin PM. [Epstein-Barr virus as a marker of biological aggressiveness in breast cancer.](https://www.ncbi.nlm.nih.gov/pubmed/21179039) Br J Cancer 2011;104:332-337.

12. [Hachana M](https://www.ncbi.nlm.nih.gov/pubmed/?term=Hachana%20M%5BAuthor%5D&cauthor=true&cauthor_uid=22024152), [Amara K](https://www.ncbi.nlm.nih.gov/pubmed/?term=Amara%20K%5BAuthor%5D&cauthor=true&cauthor_uid=22024152), [Ziadi S](https://www.ncbi.nlm.nih.gov/pubmed/?term=Ziadi%20S%5BAuthor%5D&cauthor=true&cauthor_uid=22024152), [Romdhane E](https://www.ncbi.nlm.nih.gov/pubmed/?term=Romdhane%20E%5BAuthor%5D&cauthor=true&cauthor_uid=22024152), [Gacem RB](https://www.ncbi.nlm.nih.gov/pubmed/?term=Gacem%20RB%5BAuthor%5D&cauthor=true&cauthor_uid=22024152), [Trimeche M](https://www.ncbi.nlm.nih.gov/pubmed/?term=Trimeche%20M%5BAuthor%5D&cauthor=true&cauthor_uid=22024152). Investigation of Epstein-Barr virus in breast carcinomas in Tunisia. [Pathol Res Pract.](https://www.ncbi.nlm.nih.gov/pubmed/?term=hachana++2011+EBV) 2011;207:695-700.

13. Glenn WK, Heng B, Delprado W, Iacopetta B, Whitaker NJ, Lawson JS. Epstein-Barr virus, human papillomavirus and mouse mammary tumour virus as multiple viruses in breast cancer. PLoS One 2012;7:e48788.

14. Zekri AR, Bahnassy AA, Mohamed WS, El-Kassem FA, El-Khalidi SJ, Hafez MM, et al. Epstein-Barr virus and breast cancer: epidemiological and molecular study on Egyptian and Iraqi women. J Egypt Natl Canc Inst 2012;24:123–131.

15. Khabaz MN. [Association of Epstein-Barr virus infection and breast carcinoma.](https://www.ncbi.nlm.nih.gov/pubmed/24049539) Arch Med Sci 2013;9:745-751

16. Yahia ZA, Adam AA, Elgizouli M, Hussein A, Masri MA, Kamal M, et al. Epstein Barr virus: a prime candidate of breast cancer aetiology in Sudanese patients. Infect Agent Cancer 2014;9:9.

17. Mohammadizadeh F, Zarean M, Abbasi M. [Association of Epstein-Barr virus with invasive breast carcinoma and its impact on well-known clinicopathologic parameters in Iranian women.](https://www.ncbi.nlm.nih.gov/pubmed/25161988) Adv Biomed Res. 2014 ;3:141.

18. Richardson AK, Currie MJ, Robinson BA, Morrin H, Phung Y, Pearson JF, Anderson TP, Potter JD, Walker LC. [Cytomegalovirus and Epstein-Barr virus in breast cancer.](https://www.ncbi.nlm.nih.gov/pubmed/25723522) PLoS One 2015;10:e0118989.

19. Ahmed RA_, Yussif SM Immunohistochemical detection of human cytomegalovirus, Epstein-Barr virus and human papillomavirus in invasive breast carcinoma in Egyptian women: A tissue microarray study. J Solid Tumors 2016;6:6-16

20. El-Naby NEH, Hassan Mohamed H, Mohamed Goda A, El Sayed Mohamed A. Epstein-Barr virus infection and breast invasive ductal carcinoma in Egyptian women: a single center experience. J Egypt Natl Canc Inst 2017;29:77–82.

# 21.[Fessahaye G](https://www.ncbi.nlm.nih.gov/pubmed/?term=Fessahaye%20G%5BAuthor%5D&cauthor=true&cauthor_uid=29299053), [Elhassan AM](https://www.ncbi.nlm.nih.gov/pubmed/?term=Elhassan%20AM%5BAuthor%5D&cauthor=true&cauthor_uid=29299053), [Elamin EM](https://www.ncbi.nlm.nih.gov/pubmed/?term=Elamin%20EM%5BAuthor%5D&cauthor=true&cauthor_uid=29299053), [Adam AAM](https://www.ncbi.nlm.nih.gov/pubmed/?term=Adam%20AAM%5BAuthor%5D&cauthor=true&cauthor_uid=29299053), [Ghebremedhin A](https://www.ncbi.nlm.nih.gov/pubmed/?term=Ghebremedhin%20A%5BAuthor%5D&cauthor=true&cauthor_uid=29299053), [Ibrahim ME](https://www.ncbi.nlm.nih.gov/pubmed/?term=Ibrahim%20ME%5BAuthor%5D&cauthor=true&cauthor_uid=29299053). Association of Epstein - Barr virus and breast cancer in Eritrea. [Infect Agent Cancer.](https://www.ncbi.nlm.nih.gov/pubmed/?term=fessahaye+2017+ebv) 2017;12:62.

22 . Pai T, Gupta S, Gurav M, Nag S, Shet T, Patil A, et al. Evidence for the association of Epstein-Barr virus in breast cancer in Indian patients using in-situ hybridization technique. Breast J 2018;24:16-22.

23. Kalkan A, Ozdarendeli A, Bulut Y, Yekeler H, Cobanoglu B, Doymaz MZ. [Investigation of Epstein-Barr virus DNA in formalin-fixed and paraffin- embedded breast cancer tissues.](https://www.ncbi.nlm.nih.gov/pubmed/15961939) Med Princ Pract. 2005;14:268-271.

24. [Morales-Sánchez A](https://www.ncbi.nlm.nih.gov/pubmed/?term=Morales-S%C3%A1nchez%20A%5BAuthor%5D&cauthor=true&cauthor_uid=24131889), [Molina-Muñoz T](https://www.ncbi.nlm.nih.gov/pubmed/?term=Molina-Mu%C3%B1oz%20T%5BAuthor%5D&cauthor=true&cauthor_uid=24131889), [Martínez-López JL](https://www.ncbi.nlm.nih.gov/pubmed/?term=Mart%C3%ADnez-L%C3%B3pez%20JL%5BAuthor%5D&cauthor=true&cauthor_uid=24131889), [Hernández-Sancén P](https://www.ncbi.nlm.nih.gov/pubmed/?term=Hern%C3%A1ndez-Sanc%C3%A9n%20P%5BAuthor%5D&cauthor=true&cauthor_uid=24131889), [Mantilla A](https://www.ncbi.nlm.nih.gov/pubmed/?term=Mantilla%20A%5BAuthor%5D&cauthor=true&cauthor_uid=24131889), [Leal YA](https://www.ncbi.nlm.nih.gov/pubmed/?term=Leal%20YA%5BAuthor%5D&cauthor=true&cauthor_uid=24131889), [Torres J](https://www.ncbi.nlm.nih.gov/pubmed/?term=Torres%20J%5BAuthor%5D&cauthor=true&cauthor_uid=24131889), [Fuentes-Pananá EM](https://www.ncbi.nlm.nih.gov/pubmed/?term=Fuentes-Panan%C3%A1%20EM%5BAuthor%5D&cauthor=true&cauthor_uid=24131889). No association between Epstein-Barr Virus and Mouse Mammary Tumor Virus with breast cancer in Mexican women. [Sci Rep.](https://www.ncbi.nlm.nih.gov/pubmed/?term=morales+sanchez++2013+ebv) 2013;3:2970.

25. Peng J, Wang T, Zhu H, Guo J, Li K, Yao Q, et al. Multiplex PCR/mass spectrometry screening of biological carcinogenic agents in human mammary tumors. J Clin Virol. 2014; 61: 255–259.

26. Al Hamad M, Matalka I, Al Zoubi MS, Armogida I, Khasawneh R, Al-Husaini M, Sughayer M, Jaradat S, Al-Nasser AD, Mazzanti CM. [Human mammary tumor virus, human papilloma virus, and Epstein-Barr Virus infection are associated with sporadic breast cancer metastasis.](https://pubmed.ncbi.nlm.nih.gov/33281452/) Breast Cancer (Auckl). 2020;14:1178223420976388.

27. Alinezhad F, Ahangar Oskouee M, Bannazadeh Baghi H, Tamiri Oskouee S, Esmaeili HA. [Evidence of Epstein-Barr Virus in Female Breast Cancer.](https://pubmed.ncbi.nlm.nih.gov/33748013/)

Iran J Public Health. 2021;50(2):425-427.

Studies of Epstein Barr virus and breast cancer by Kalkan et al 2005, Morales-Sanchez et al 2013, Peng et al 2014, Sharifpour 2019 [23,24,25, 27] have not been included in Table 3 because infiltrating lymphocytes could not be excluded.

| Study | Location | Breast non-cancer controls | BLV positive breast cancer | BLV positive normal benign breast | Statistical significance |
| --- | --- | --- | --- | --- | --- |
| Giovanna 2013 [1] | Columbia | Benign breast | 19/53 36% | 24/53 45% | 0.682 ns |
| Buehring 2015 [2] | US | Normal breast | 67/114 59% | 30/104 29% | 0.001 |
| Zhang 2016 [3] | China | Blood | 0/91 0% | 0/100 0% |  |
| Buehring  2017 [4] | Australia | Normal breast | 40/50 80% | 19/46 41% | 0.001 |
| Baltzell 2017 [5] | US | Benign breast | 35/61 57% | 20/103 20% | 0.059 ns |
| Khalilian 2019 [6] | Iran | Benign breast | 57/172 30% | 5/28 8% | 0.001 |
| Schwingel 2019 [7] | Brazil | Normal breast | 22/72 30.5% | 10/72 13.9% | 0.017 |
| Delamelina 2020 [8] | Brazil | Normal breast | 47/49 96% | 23/39 59% | 0.001 |

**Supplementary Table 4. Identification of Bovine leukemia virus in human breast cancer (Case control studies)**

ns = not significant at 0.05 level

**References**

1. Giovanna M, Carlos UJ, María UA, Gutierrez MF. Bovine Leukemia Virus Gene Segment Detected in Human Breast Tissue Open J Med Microbiol 2013;3:84-90

2. [Buehring GC](https://www.ncbi.nlm.nih.gov/pubmed/?term=Buehring%20GC%5BAuthor%5D&cauthor=true&cauthor_uid=26332838), [Shen HM](https://www.ncbi.nlm.nih.gov/pubmed/?term=Shen%20HM%5BAuthor%5D&cauthor=true&cauthor_uid=26332838), [Jensen HM](https://www.ncbi.nlm.nih.gov/pubmed/?term=Jensen%20HM%5BAuthor%5D&cauthor=true&cauthor_uid=26332838), [Jin DL](https://www.ncbi.nlm.nih.gov/pubmed/?term=Jin%20DL%5BAuthor%5D&cauthor=true&cauthor_uid=26332838), [Hudes M](https://www.ncbi.nlm.nih.gov/pubmed/?term=Hudes%20M%5BAuthor%5D&cauthor=true&cauthor_uid=26332838), [Block G](https://www.ncbi.nlm.nih.gov/pubmed/?term=Block%20G%5BAuthor%5D&cauthor=true&cauthor_uid=26332838). Exposure to Bovine Leukemia Virus Is Associated with Breast Cancer: A Case-Control Study. [PLoS One.](https://www.ncbi.nlm.nih.gov/pubmed/?term=buehring+g+2015) 2015;10:e0134304..

3. Zhang R, Jiang J, Sun W, Zhang J, Huang K, Gu X, Yang Y, Xu X, Shi Y, Wang C. [Lack of association between bovine leukemia virus and breast cancer in Chinese patients.](https://www.ncbi.nlm.nih.gov/pubmed/27724949) Breast Cancer Res 2016;18:101.

4. [Buehring GC](https://www.ncbi.nlm.nih.gov/pubmed/?term=Buehring%20GC%5BAuthor%5D&cauthor=true&cauthor_uid=28640828), [Shen H](https://www.ncbi.nlm.nih.gov/pubmed/?term=Shen%20H%5BAuthor%5D&cauthor=true&cauthor_uid=28640828), [Schwartz DA](https://www.ncbi.nlm.nih.gov/pubmed/?term=Schwartz%20DA%5BAuthor%5D&cauthor=true&cauthor_uid=28640828), [Lawson JS](https://www.ncbi.nlm.nih.gov/pubmed/?term=Lawson%20JS%5BAuthor%5D&cauthor=true&cauthor_uid=28640828). Bovine leukemia virus linked to breast cancer in Australian women and identified before breast cancer development. [PLoS One.](https://www.ncbi.nlm.nih.gov/pubmed/28640828) 2017;12:e0179367.

5. [Baltzell KA](https://www.ncbi.nlm.nih.gov/pubmed/?term=Baltzell%20KA%5BAuthor%5D&cauthor=true&cauthor_uid=29266207), [Shen HM](https://www.ncbi.nlm.nih.gov/pubmed/?term=Shen%20HM%5BAuthor%5D&cauthor=true&cauthor_uid=29266207), [Krishnamurthy S](https://www.ncbi.nlm.nih.gov/pubmed/?term=Krishnamurthy%20S%5BAuthor%5D&cauthor=true&cauthor_uid=29266207), [Sison JD](https://www.ncbi.nlm.nih.gov/pubmed/?term=Sison%20JD%5BAuthor%5D&cauthor=true&cauthor_uid=29266207), [Nuovo GJ](https://www.ncbi.nlm.nih.gov/pubmed/?term=Nuovo%20GJ%5BAuthor%5D&cauthor=true&cauthor_uid=29266207), [Buehring GC](https://www.ncbi.nlm.nih.gov/pubmed/?term=Buehring%20GC%5BAuthor%5D&cauthor=true&cauthor_uid=29266207). Bovine leukemia virus linked to breast cancer but not coinfection with human papillomavirus: Case-control study of women in Texas. [Cancer.](https://www.ncbi.nlm.nih.gov/pubmed/?term=Baltzell+2017++bovine) 2018;124:1342-1349.

6. Khalilian M, Hosseini SM, Madadgar O. [Bovine leukemia virus detected in the breast tissue and blood of Iranian women.](https://www.ncbi.nlm.nih.gov/pubmed/31252065) Microb Pathog 2019;135:103566

7. Schwingel D, Andreolla AP, Erpen LMS, Frandoloso R, Kreutz LC. [Bovine leukemia virus DNA associated with breast cancer in women from South Brazil.](https://www.ncbi.nlm.nih.gov/pubmed/30814631) Sci Rep 2019;9:2949.

8. Delarmelina E, Buzelin MA, Souza BS, Souto FM, Bicalho JM, Câmara RJF, Resende CF, Bueno BL, Victor RM, Galinari GCF, Nunes CB, Leite RC, Costa ÉA, Reis JKPD. [High positivity values for bovine leukemia virus in human breast cancer cases from Minas Gerais, Brazil.](https://pubmed.ncbi.nlm.nih.gov/33017448/) PLoS One. 2020;15:e0239745.
